# Detection of reproducible liver cancer specific ligand-receptor signaling in blood

**DOI:** 10.3389/fbinf.2024.1332782

**Published:** 2025-01-09

**Authors:** Aram Safrastyan, Damian Wollny

**Affiliations:** ^1^ RNA Bioinformatics and High Throughput Analysis, Friedrich Schiller University Jena, Jena, Germany; ^2^ Genetics and Epigenetics of Aging, Leibniz Institute on Aging-Fritz Lipmann Institute (FLI), Jena, Germany; ^3^ Department of Evolutionary Genetics, Max Planck Institute for Evolutionary Anthropology, Leipzig, Germany

**Keywords:** liquid biopsy, liver cancer, cell-cell interaction, single-cell RNA sequencing, cell-free RNA, bioinformatics

## Abstract

Cell-cell communication mediated by ligand-receptor interactions (LRI) is critical to coordinating diverse biological processes in homeostasis and disease. Lately, our understanding of these processes has greatly expanded through the inference of cellular communication, utilizing RNA extracted from bulk tissue or individual cells. Considering the challenge of obtaining tissue biopsies for these approaches, we considered the potential of studying cell-free RNA obtained from blood. To test the feasibility of this approach, we used the BulkSignalR algorithm across 295 cell-free RNA samples and compared the LRI profiles across multiple cancer types and healthy donors. Interestingly, we detected specific and reproducible LRIs particularly in the blood of liver cancer patients compared to healthy donors. We found an increase in the magnitude of hepatocyte interactions, notably hepatocyte autocrine interactions in liver cancer patients. Additionally, a robust panel of 30 liver cancer-specific LRIs presents a bridge linking liver cancer pathogenesis to discernible blood markers. In summary, our approach shows the plausibility of detecting liver LRIs in blood and builds upon the biological understanding of cell-free transcriptomes.

## 1 Introduction

Cancer remains one of the most pressing healthcare challenges globally, being the second leading cause of death worldwide (GBD, 2015 Mortality and Causes of Death Collaborators et al., 2016). Numerous studies have shown that early cancer detection significantly improves the survival rate, emphasizing the importance of improved detection methods (Crosby et al., 2022). A promising method for minimally invasive yet highly informative diagnostics is liquid biopsy. This methodology focuses on analyzing body fluids - primarily blood - utilizing various omics techniques such as proteomics, genomics, and, notably, transcriptomics (Heitzer et al., 2019). In particular, the analysis of cell-free RNAs - which are RNAs that have exited the cells either as a result of cell death or active secretion and are shed into the bloodstream from around the body (Jin et al., 2023; Vorperian et al., 2022) - is increasingly promising (Cabús et al., 2022) due to the fact that transcriptomic signatures can reveal tissue and cell-type specificity which would greatly aid diagnostics (Jin et al., 2023; Koh et al., 2014; Vong et al., 2021; Vorperian et al., 2022; Zaporozhchenko et al., 2018). Beyond diagnostics, cell-free RNA can in principle offer many insights into cellular processes of cells throughout the body since RNAs are constantly being shed into the bloodstream (Ibarra et al., 2020; Zaporozhchenko et al., 2018). Yet, to which extent we can learn about inter- and intracellular processes solely by investigating the limited subset of cell transcriptomes that enter into the bloodstream is currently unknown.

In recent years, numerous approaches have focussed on mapping ligand-receptor interactions as they are crucial for comprehending cellular responses and intercellular communication networks (Cabello-Aguilar et al., 2020; Dimitrov et al., 2022; Efremova et al., 2020; Lu et al., 2022; Jin et al., 2021; Wangzhou et al., 2021). In particular, single-cell RNA sequencing (scRNA-seq) technology enables the measurement of ligand and receptor expression across various cell types, facilitating the systematic decoding of intracellular communication for the maintenance of homeostasis but also in cancerogenesis (Ghoshdastider et al., 2021; Ramilowski et al., 2015; Zhou et al., 2017). In order to experimentally gain these insights, however, tissue biopsies are needed which are difficult to extract and only provide a snapshot in time. Thus, we asked the question as to which degree one could observe LRI differences between cancer and normal tissue solely by exploring the cell-free RNA found in the blood of patients and healthy donors.

In this proof-of-concept study, using the BulkSignalR algorithm (Villemin et al., 2023), we queried LRIs in close to 300 blood samples, including those from cancer patients and healthy donors. In our analysis of all the samples, liver cancer samples notably distinguished themselves. We showed not only the possibility of inferring relevant LRIs from cell-free transcriptomes in blood from liver cancer patients but also highlighted an increase in the number of interactions associated with hepatocytes in these patients. Furthermore, we curated a panel of 30 highly robust LRIs in the cell-free transcriptome specific to liver cancer. Within this panel, we find previously documented liver- and liver cancer-relevant marker gene ligands SERPINC1 and GPC3 LRIs to be specific and unique to liver cancer blood samples and thus serve as potentially potent biomarkers.

## 2 Materials and methods

### 2.1 Research strategy

The central aim of the study was the investigation of the possibility of deriving ligand-receptor interactions (LRI) and cell-cell interactions (CCI) from blood cell-free transcriptomes. The software tool used to analyze LRIs in blood cell-free transcriptomes was the recently developed BulkSignalR (Villemin et al., 2023), which detects directional LRIs from bulk RNA-seq data and has specific features that differentiate it from similar tools designed to analyze single-cell RNA datasets, such as CellPhoneDB (Efremova et al., 2020) or SingleCellSignalR (Cabello-Aguilar et al., 2020). The latter tools deal with populations of single-cell types where it is evident which cell types express the ligand and the corresponding receptor. Therefore, the output of CellPhoneDB and similar tools include directional CCI corresponding to different directional LRIs. In this instance, the main challenge is designating a method for identifying biologically relevant LRIs, which, for example, CellPhoneDB achieves by randomly permutating the cell type labels of all cells and calculating the average ligand and receptor expressions in each cell type, which yields a null distribution for each LRI responsible for a particular CCI (Efremova et al., 2020). The null distribution is used to calculate a p-value describing the enrichment of each ligand-receptor pair in the cell type populations being analyzed and, as such, allows to prioritize the more cell-type specific interactions (Efremova et al., 2020).

On the other hand, bulk RNA-seq data does not possess cell-type level information and represents an aggregation of gene expressions across different cell types. Therefore, BulkSignalR achieves the removal of false positive LRIs by calculating p-values from null distributions of Spearman correlation coefficients between ligand-receptor pairs in each randomized gene expression dataset generated from the input dataset (Villemin et al., 2023). Another consideration is that while BulkSignalR is capable of providing directional LRIs, it is unable to produce CCIs due to the previously outlined lack of cell-type level information in bulk RNA-seq data. A potential workaround for this limitation is the leveraging of single-cell RNA-seq data, which can provide information about CCIs, and it is this strategy - the alignment of LRIs derived from bulk and single-cell RNA-seq datasets - that we employed in the current study to analyze CCIs from blood cell-free transcriptomes.

### 2.2 Detection of LRIs

We used publicly available RNA-seq datasets generated by Chen et al. (2022) and Zhu et al. (2021) to study the LRIs in blood cell-free transcriptomes. The count matrices of raw reads were downloaded from Gene Expression Omnibus (GEO) with the ascension numbers GSE174302 and GSE142987. In total, 295 blood samples from five types of solid tumors and healthy donors were analyzed (Table 1). Liver cancer (LC) blood samples (n = 62) were mainly drawn from hepatocellular carcinoma (HCC) patients, with only eight samples drawn from intrahepatic cholangiocarcinoma (ICC) patients (Chen et al., 2022; Zhu et al., 2021). Additionally, more than 60% of liver cancer patients had chronic hepatitis B (CHB) infection (Chen et al., 2022). In order to infer LRIs, the count matrices were separately (per biological condition) prepared for subsequent analysis using the function “prepareDataset” from the R (version 4.1.2) (R Core Team, 2021) package BulkSignalR (version 0.0.9) (Villemin et al., 2023). Next, the function “learnParameters” from the package BulkSignalR was employed to estimate the statistical model parameters and finally, with the function “initialInference” from the package BulkSignalR LRIs are inferred and stored in a BSRInference object. Dataframes of inferred LRIs were extracted from the BSRInference objects using the function “LRInter” from the package BulkSignalR. A threshold of 0.1% FDR was applied, as described in the original study (Villemin et al., 2023).

**TABLE 1:** Main characteristics of the RNA-seq datasets used in the study. The values indicate the number of donor/patient samples in each dataset. Accession numbers refer to the Gene Expression Omnibus (GEO) database.

Dataset	HD	LC	STAD	LUAD	CRC	ESCA	Accession number	Reference
Chen et al. (2022)	46	27	37	35	54	31	GSE174302	Chen et al. (2022)
Zhu et al. (2021)	30	35	NA	NA	NA	NA	GSE142987	Zhu et al. (2021)
Single-cell RNA-seq	NA	17,392 cells/5 patients	NA	NA	NA	NA	GSE149614	Lu et al. (2022)
Bulk RNA-seq	NA	35	NA	NA	NA	NA	GSE124535	Jiang et al. (2019), Zeng et al. (2020)

HD, healthy donor; LC, liver cancer; STAD, stomach adenocarcinoma; LUAD, lung adenocarcinoma; CRC, colorectal cancer; ESCA, esophageal cancer.

A count matrix of bulk RNA-seq dataset of CHB HCC tissue samples (Jiang et al., 2019; Zeng et al., 2020) was downloaded from GEO under the ascension number GSE124535. The count matrix contained FPKM values of 35 HCC liver tissue samples and was used to infer LRIs with the R package BulkSignalR as described with the parameter “normalize = FALSE” in the “prepareDataset” function. In order to get only high-confidence LRIs, a threshold of 0.1% FDR and a minimum ligand-receptor correlation value of 0.5 were applied.

Finally, a single-cell RNA-seq dataset of HCC patient liver tissue samples (Lu et al., 2022) was downloaded from the GEO database under the ascension number GSE149614. After selecting cells extracted from primary tumor sites of CHB patients, there remained 17,392 cells from five patients. To assign cell types we used the R Bioconductor (Huber et al., 2015) package SingleR (version 1.8.0) (Aran et al., 2019) and a healthy liver single-cell RNA-seq dataset (MacParland et al., 2018) as reference. The latter was downloaded from the GEO database and included log2CPM values of 8,444 cells from five healthy donor liver tissues. After collapsing the annotations for hepatocytes, macrophages, T cells and liver sinusoidal endothelial cells (LSECs), 11 cell-type annotations remained and, together with the liver cancer scRNA-seq data were used as input for the “SingleR” function of the SingleR package with the parameter “de.method = “wilcox”“. Then, the annotated liver cancer scRNA-seq data was normalized with the function “NormalizeData” of the Seurat R package (version 4.3.0.1) (Hao et al., 2021). Finally, LRIs were inferred from the annotated and normalized HCC single-cell RNA-seq data using the function “liana_wrap” from the LIANA R package (version 0.1.12) (Dimitrov et al., 2022) with the parameter “resource = “LRdb”” as the package BulkSignalR also uses the database LR*db* (Cabello-Aguilar et al., 2020). In order to acquire only high-confidence LRIs, we applied a threshold of 0.05 for the CellPhoneDB *p*-values (Dimitrov et al., 2022; Efremova et al., 2020) and a threshold of 0.5 for the correlations between ligands and receptors (Dimitrov et al., 2022; Villemin et al., 2023).

The LRIs inferred from Chen et al. liquid biopsy datasets were used as input for the “UpsetR” function from the UpsetR R package (version 1.4.0) (Conway et al., 2017) to generate UpSet plots. Only the first ten intersections ordered by size were shown for visualization purposes. The intersection between liver cancer samples from Chen et al. and Zhu et al. datasets was visualized using the “venn” function from the R package ggvenn (version 0.1.10) (Yan, 2023) with the parameter “auto_scale = T”.

The total number of LRIs identified in Chen et al. and Zhu et al. datasets was visualized using the R package ggplot2 (version 3.4.3) (Hadley, 2016) and ggpubr (version 0.6.0) (Kassambara, 2023). The relationship between tissue bulk and single-cell RNA-seq LRIs was visualized with the “ggvenn” function from the ggvenn R package with the parameter “auto_scale = TRUE”.

### 2.3 Inference of cellular interactions

In order to associate the identified LRIs in the cell-free transcriptome with source and target cell types, we used the scRNA-seq results where each identified LRI was assigned to a source and target cell type found in the liver. First, the healthy donor blood LRIs were removed from the lists of LRIs identified in the blood of cancer patients. Then, the scRNA-seq LRI dataframe was filtered by LRIs found in each cell-free data type. Then, in each resulting dataframe the number of interactions between cell types was calculated and the top ten most abundant interactions were selected. Finally, the selected cell interactions were used to construct cellular networks with the R package circlize (version 0.4.14) (Gu et al., 2014) using the function “chordDiagramFromMatrix”. Hepatocyte autocrine interactions were highlighted where present with a dashed line.

### 2.4 Generation of an LC-specific and reproducible panel of LRIs

To generate a panel of LC-specific and reproducible LRIs, we first excluded every LRI found in liver cancer cell-free dataset of Chen et al. that could also be found in Chen et al. healthy donor, esophageal cancer, stomach adenocarcinoma, colorectal cancer and lung adenocarcinoma cell-free datasets. Then, we intersected the remaining LRIs with those found in Zhu et al. liver cancer cell-free dataset and finally excluded LRIs found also in Zhu et al. healthy donor cell-free dataset. The final list included 30 LRIs which were used to generate a heatmap with corresponding average expressions in the scRNA-seq dataset of Lu et al. (2022), which were calculated using the “AverageExpression” function in the Seurat R package. Considering that LRIs may be associated with multiple biological pathways, we visualized the largest downstream pathways per LRI.

To further contextualize the identified LRIs, the online portal CITE (Crosstalk Interactions within Tumor microenvironment; https://cite.genome.sg/) (Ghoshdastider et al., 2021) was used to acquire the estimated Relative Crosstalk (RC) score for each LRI in HCC. RC scores represent the relative concentration of LR complexes in cancer and stromal cell compartments in the tumor microenvironment and the directionality of interactions between compartments (Ghoshdastider et al., 2021). The portal contained information about 11 of the identified 30 LRIs and after retrieving the RC scores, they were used to generate a heatmap using the function “pheatmap” from the R package pheatmap (version 1.0.12) (Kolde, 2019).

In order to visualize the expression patterns of the ligands SERPINC1 and GPC3 across tumor tissues, the online portal GEPIA2 (http://gepia2.cancer-pku.cn/) was employed. After retrieving the expression values, they were used for visualization with the packages ggplot2 and ggpubr.

Unless expressly specified, all the software tools were used with default settings.

## 3 Results and discussion

### 3.1 Extensive LRI detection in liver cancer cell-free transcriptome

**FIGURE 1:**
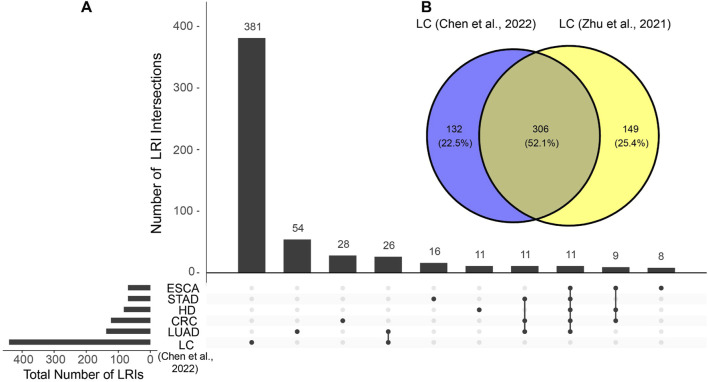
Similarities and differences of identified ligand-receptor interactions (LRIs) between cell-free RNA datasets. **(A)** LRI intersections in the cell-free RNA datasets from Chen et al. Only the ten largest intersections are shown. **(B)** LRI intersection between cell-free liver cancer (LC) datasets of Chen et al. and Zhu et al. HD, healthy donor; STAD, stomach adenocarcinoma; LUAD, lung adenocarcinoma; CRC, colorectal cancer; ESCA, esophageal cancer.

To evaluate the possibility of detecting ligand-receptor interactions (LRIs) in the cell-free transcriptome in blood, we utilized the BulkSignalR package and tested it using 295 liquid biopsy blood samples from healthy donors, liver cancer, esophageal cancer, stomach adenocarcinoma, colorectal cancer and lung adenocarcinoma sourced from the publicly available datasets of Chen et al. (2022) and Zhu et al. (2021). We detected statistically significant LRIs in both datasets, with numbers ranging from 67 to 455 (Supplementary Figure S1A; Supplementary File S1A). Notably, we found in both cell-free liver cancer datasets a markedly higher number of total and unique LRIs compared to other cell-free RNA samples from other types of cancer (Supplementary Figure S1A; Figure 1A), underscoring the pronounced increase in tissue signal presence in blood during liver cancerogenesis. Most of the LRIs identified in cell-free RNA liver cancer data were reproducible in both datasets (Figure 1B), reducing the likelihood of them being attributed to random noise. This unique feature of liver cancer results can be explained by the pronounced contribution of the liver to the cell-free transcriptome (Larson et al., 2021) which means that any pathological changes within the liver are prominently reflected in blood samples (Morlion et al., 2023; Safrastyan et al., 2023; Vong et al., 2021). Earlier studies have highlighted detectable alterations in the cell-free transcriptome during liver diseases, with particular shifts in cell-type signals (Vorperian et al., 2022). Hence, this finding indicates that liver cancer is a promising use case for studying LRI changes during carcinogenesis.

### 3.2 Increase in the number of hepatocyte interactions in liver cancer blood samples

One of the main advantages of LRIs is the ability to infer cell-cell interactions and cellular networks. We decided to infer changes in cell-cell interactions of liver cancer patients and healthy donors from the blood samples by leveraging a single-cell RNA-sequencing (scRNA-seq) dataset of liver cancer patient tissue samples (Lu et al., 2022). Our analysis of LRIs in the scRNA-seq data yielded 1,120 LRIs which were associated with 11 source and target cell types found in the liver (Supplementary Figure S1B; Supplementary File S1). Additionally, considering previous bulk RNA-sequencing findings which highlighted lowly expressed LRIs not captured by scRNA-seq data analysis (Villemin et al., 2023), we also identified LRIs in bulk RNA-sequencing data of liver cancer tissue samples to achieve a more comprehensive collection of LRIs. (Supplementary Figure S1B; Supplementary File S1) (Jiang et al., 2019; Zeng et al., 2020). Consistent with a prior observation (Villemin et al., 2023), a greater number of LRIs were detected in scRNA-seq data, with bulk RNA-sequencing contributing a modest number of unique LRIs (Supplementary Figure S1B).

**FIGURE 2:**
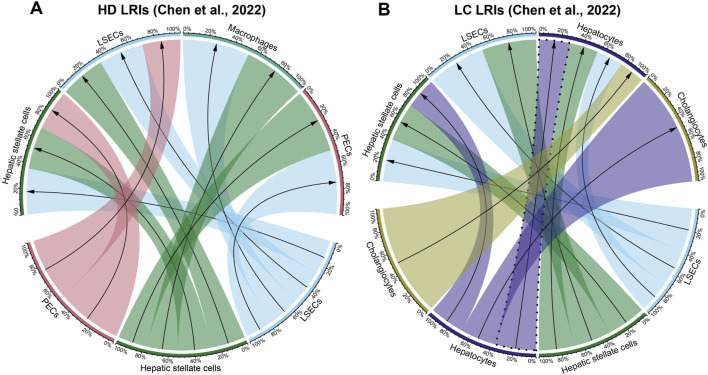
Cellular interactions in cell-free RNA datasets of Chen et al. **(A)** Healthy donor (HD) and **(B)** liver cancer (LC) datasets. Visualized are the ten most abundant cell-cell interactions found in each dataset. Hepatocyte autocrine interactions are highlighted with a dashed line. Percentages represent the proportion of each cell-cell interaction for each source and target cell types. The direction of the arrows corresponds to the interaction starting from the ligand on the source cell type (bottom half) to the receptor on the target cell type (top half). The full list of detected cell-cell interactions per condition corresponding to the ligand-receptor interactions described in Supplementary File S1 is available in Supplementary File S2.

We then visualized the top ten most abundant cell-cell interactions found in the scRNA-seq results for each cell-free RNA dataset. In both Chen et al. and Zhu et al. cell-free RNA liver cancer datasets, our analysis of the ten most abundant cell-cell interactions (Figure 2; Supplementary Figure S2; Supplementary File S2) showed strong hepatocyte signaling, both as a source and a target (Figure 2B; Supplementary Figure S2F). In contrast, hepatocyte signaling was less pronounced and ligands originating from hepatocytes were absent in healthy donor (Figure 2A; Supplementary Figure S2E) and other cancer blood samples (Supplementary Figures S2A–D). This aligns with prior studies describing an increase in hepatocyte signaling in the blood of liver cancer patients (Morlion et al., 2023; Vong et al., 2021).

Another noteworthy observation was the detection of an abundant hepatocyte autocrine interaction in the liquid biopsy liver cancer in contrast to healthy control datasets (Figure 2B; Supplementary Figure S2F). This finding confirms observations by previous studies indicating that the increase in autocrine hepatocyte signaling is a strong signal for liver carcinogenesis that can also be detected in the cell-free transcriptome (Ghoshdastider et al., 2021; Lu et al., 2022; Tummala et al., 2017).

### 3.3 In-depth analysis of highly robust liver cancer LRIs

**FIGURE 3:**
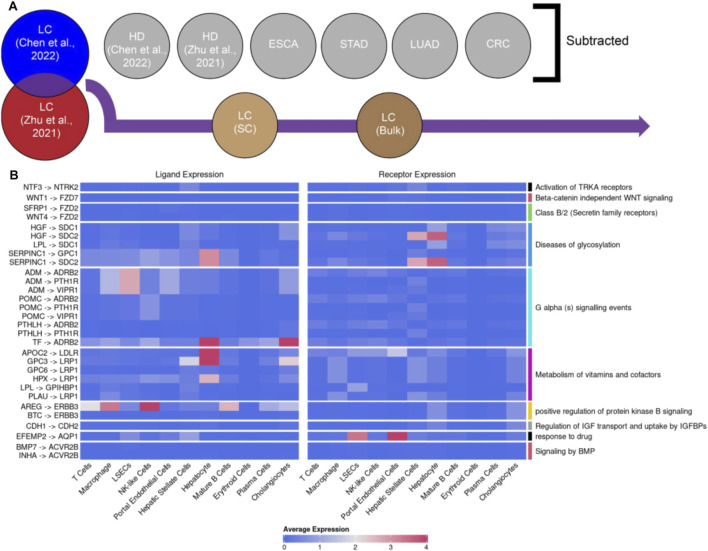
Curation of a panel of reproducible liver cancer specific ligand-receptor interactions (LRIs). **(A)** Schematic overview of the pipeline. Briefly, from the common LRIs between cell-free LC datasets of Chen et al. and Zhu et al. we excluded LRIs found in cell-free healthy donor (HD), esophageal cancer (ESCA), stomach adenocarcinoma (STAD), lung adenocarcinoma (LUAD) and colorectal cancer (CRC) datasets. Subsequently, LRIs not found in either liver cancer single-cell of bulk RNA-seq datasets were excluded as well. **(B)** Visualization of the identified panel of 30 LRIs and the largest regulated downstream pathway. The average expression for each ligand and receptor part of the 30 LRI panel per cell type in the LC scRNA-seq dataset was calculated by averaging the exponentiated log-normalized counts, which were then transformed back to the natural logarithmic scale. The LRIs with 0 average expression across all cell types were identified in the bulk RNA-seq but not scRNA-seq data.

In order to more deeply understand the biological relevance of liver cancer-specific LRIs, our goal was to distill highly robust LRI signals. To achieve that, we created a panel of liver cancer-specific and reproducible LRIs, from the list of LRIs shared between Chen et al. and Zhu et al. cell-free liver cancer datasets. In this panel, we excluded all LRIs found in other cell-free datasets to maximize the specificity towards liver cancer signals (Figure 3A). Next, to ensure that only LRIs remained which were previously reported to be present in liver cancer tissue, we filtered out the LRIs not found in either single-cell or bulk liver cancer RNA-seq datasets (Figure 3A). The final list consisted of 30 LRIs involving 22 ligands and 16 receptors (Figure 3B; Supplementary File S3). Notably, many of the identified ligands and receptors were previously associated with liver cancer. Among the candidates that were found, SERPINC1, GPC3 and the receptor ERBB3 were particularly noteworthy. SERPINC1 has a pronounced specificity to the liver and gallbladder (Xu et al., 2021) and its expression increases further in hepatocellular carcinoma (HCC) (Xu et al., 2021) and decreases during cholangiocarcinoma (ICC) (Supplementary Figure S3A) (Li et al., 2019; Wang et al., 2006) which also indicates its potential as a marker to differentiate liver cancer subtypes. GPC3 is an oncofetal marker for the liver whereas in the healthy adult liver little GPC3 expression has been detected (Morford et al., 2007; Zhou et al., 2018). Yet, upon cancerogenesis, the ligand is highly expressed in HCC (Supplementary Figure S3B) (Morford et al., 2007; Zhou et al., 2018). GPC3 promotes cancerogenesis via activation of the Wnt/β-catenin signaling pathway and, hence, presents itself as a potential therapeutic target (Kolluri and Ho, 2019; Shih et al., 2020). Finally, another noteworthy receptor from the list is ERBB3 (HERT3) which is upregulated in chronic hepatitis B (CHB) but not hepatitis C induced HCC (Buta et al., 2016) corresponding to the liquid biopsy liver cancer sample composition we employed (>60% CHB) (Chen et al., 2022).

Similarly, most of the downstream pathways regulated by the detected LRIs (Figure 3B) have previously documented relevance to liver pathologies (Ehata and Miyazono, 2022; Gao et al., 2021; Li et al., 2021; Mehta et al., 2015; Wang et al., 2021; Whittaker et al., 2010). For example, the detection of a pathway involved in the metabolism of vitamins and cofactors aligns well with the prominent role the liver plays in these processes, the dysregulation of which is also commonly observed during liver pathologies (Fang et al., 2019; Licata et al., 2021; Paganoni et al., 2021; Raza et al., 2021; Roberts and Sarkar, 2008). Similarly, the observation of downstream pathways relating to WNT and protein kinase B (AKT) signaling is in line with the observation of ectopic activation of WNT signaling being one of the main hallmarks of HCC (Khalaf et al., 2018; Kolluri and Ho, 2019; Perugorria et al., 2019; Wang et al., 2019) and AKT acting as a key regulator of HCC progression (Khalaf et al., 2018; Nicholson and Anderson, 2002; Tian et al., 2023).

In addition, we endeavored to further contextualize the identified LRIs by using the CITE platform which assigns Relative Crosstalk (RC) scores to LRIs based on the expression patterns of the LRIs in the tumor microenvironment (Ghoshdastider et al., 2021). Of the 30 LRIs identified by us, 11 had RC scores assigned by CITE in HCC (Supplementary Figure S4), with the vast majority having low RC scores in the normal cell compartment which suggests a higher association of these LRIs with cancer. The majority of the LRIs also had a high RC score in the stromal compartment, which includes autocrine LRIs between stromal cells and paracrine LRIs between stromal and cancer compartments, in line with previous findings (Ghoshdastider et al., 2021). Here, the INHA-ACVR2B LRI stands out given its high cancer compartment autocrine RC score which is in line with a prior observation of ACVR2B interactions being very prevalent in cancer-cancer communication across different tumor types (Ghoshdastider et al., 2021) and lends further support to our observation of a potential hepatocyte autocrine signaling. Together, these findings suggest that the cell-free RNA signals in the blood of liver cancer patients can be utilized to indirectly gain insights into changes in ligand-receptor interactions during liver carcinogenesis.

## 4 Conclusion

Our findings reveal the ability to detect LRIs in the blood of healthy donors and patients across five solid tumor types, with a marked presence in liver cancer patients, underscoring the dominant signaling of the liver during pathological transformations. The reproducibility of these LRIs was affirmed by cross-referencing two liver cancer patient datasets, establishing the authenticity and robustness of our methodology. Additionally, an analysis of a liver cancer single-cell RNA-seq dataset spotlighted a surge in hepatocyte interactions, especially hepatocyte autocrine interactions, aligning with previous observations of elevated hepatocyte signaling in liver cancer patient blood (Morlion et al., 2023; Safrastyan et al., 2023; Vong et al., 2021).

A curated panel of 30 LRIs specific to liver cancer was formulated, several of which have known associations with LC, including noteworthy ligands like SERPINC1, GPC3, and receptors such as ERBB3. Our further analysis of the expression patterns of 11 select LRIs within the liver cancer tumor microenvironment through the CITE platform, showed that the majority of the LRIs exhibited amplified activity within liver cancer tissues relative to healthy liver with a pronounced shift towards stromal cell interactions, reinforcing the role of the stromal compartment in cancerogenesis. One LRI, INHA-ACVR2B, presented a strong association with liver cancer autocrine interactions, suggesting avenues for targeted therapeutic interventions.

It should also be noted that this proof-of-concept study will require further validation via the incorporation of larger and more diverse datasets. In particular, technical variability and lack of reproducibility are a recognized issue of cell-free RNA studies (Cabús et al., 2022), which can potentially adversely affect the results. Here, we have aimed to mitigate these issues by reproducing our major findings in two independent liver cancer cell-free RNA datasets, which significantly reduces the risk of detecting erroneous LRI enrichment. Nevertheless, further studies are required - including the inclusion of additional scRNA-seq datasets - in order to fully elucidate and verify the nature of the identified LRIs and CCIs in blood cell-free RNA data.

In conclusion, our study showcases the possibility of extracting significant LRI signals from liver cancer cell-free transcriptomic blood samples. It also accentuates the merits of liquid biopsy for liver cancer studies by constructing cellular networks that offer a broader understanding of the target tissue.

## Data Availability

Publicly available datasets were analyzed in this study. This data can be found here: https://www.ncbi.nlm.nih.gov/geo/query/acc.cgi?acc=GSE174302 https://www.ncbi.nlm.nih.gov/geo/query/acc.cgi?acc=GSE142987 https://www.ncbi.nlm.nih.gov/geo/query/acc.cgi?acc=GSE149614 https://www.ncbi.nlm.nih.gov/geo/query/acc.cgi?acc=GSE124535.
